# Occupational hypersensitivity pneumonia

**DOI:** 10.5578/tt.20239911

**Published:** 2023-03-10

**Authors:** T. AKKALE, T. SARI, C. ŞİMŞEK

**Affiliations:** 1 Clinic of Occupational Medicine, Atatürk Chest Diseases and Thoracic Surgery Training and Research Hospital, Ankara, Türkiye

**Keywords:** Hypersensitivity pneumonia, interstitial lung disease, occupational exposure, occupational hypersensitivity pneumonia, interstisyel akciğer hastalığı, mesleksel maruziyet, mesleksel hipersensitivite pnömonisi

## Abstract

**ABSTRACT:**

**Occupational hypersensitivity pneumonia:**

Hypersensitivity pneumonitis (HP) is an immunological lung disease that affects individuals who are sensitive and susceptible to occupational and environmental exposures. While clinical and radiological findings may resemble other interstitial lung diseases, identifying the causative agents can aid in the differential diagnosis. However, this can be challenging and may result in delayed diagnosis and poor prognosis. A gold standard test for diagnosis is currently unavailable, and therefore, a multidisciplinary approach involving a clinician, radiologist, and pathologist is necessary. Avoiding exposure is the first step in treatment, with immunosuppressive therapeutics also being used. Antifibrotic agents show promise for future treatment approaches. Despite recent advancements in data and guidelines, knowledge about managing occupational HP remains limited. This review provides a summary of the epidemiological, clinical, and radiological findings, as well as diagnostic and treatment principles of occupational HP based on current literature.

## Abstract (tr)

**Mesleksel hipersensitivite pnömonis:**

Hipersensitivite pnömonisi (HP) hassas veya duyarlanmış kişilerde ev içi, mesleksel veya çevresel antijenlerin tekrarlayan maruziyetleri sonucu gelişen immünolojik bir akciğer hastalığıdır. HP klinik ve radyolojik olarak diğer interstisyel akciğer hastalıkları ile benzer özelliklere sahip olsa da HP’ye neden olan etkenin tespit edilmesi tanıyı kolaylaştırmaktadır. Etkenin tanımlanamaması tanıyı geciktirmekte ve hastalığın prognozunu olumsuz etkilemektedir. Tanıda altın standart bir test olmaması nedeniyle klinisyen, radyolog ve patoloğun olduğu multidisipliner yaklaşımla tanı konulmaktadır. Etkenden uzaklaşma tedavinin temel basamağı olmakla birlikte immünsupresif tedavi de kullanılmaktadır. Antifibrotik ajanlar da tedavide gelecek vadetmektedir. HP’nin değerlendirilmesi konusunda yeni yaklaşımlar öneren son verilere ve kılavuzlara rağmen halen mesleksel HP’nin yönetiminde önemli bilgi eksiklikleri bulunmaktadır. Bu derlemede güncel bilgiler doğrultusunda mesleksel HP’nin epidemiyolojisi, klinik ve radyolojik bulguları, tanı ve tedavi prensipleri anlatılmaktadır

## Introduction

Hypersensitivity pneumonia (HP) is an immunological lung disease that causes lymphocytic and often granulomatous inflammation in the peripheral airways, alveoli, and interstitial tissue, that develops due to organic or low molecular weight chemicals ( 1 ). Its cause of origin is most commonly home or workplace. HP that develops due to exposure factors in the workplace is defined as occupational HP. The prognosis of the disease can be improved by identifying exposure sources in the workplace and avoiding the causative agent ( 2 , 3 ). Identifying the disease as occupational and reporting it can aid in disease control and protect healthy individuals in the same workplace.

The HP findings were first encountered in farmers in the 18th century; however, it was first defined as a disease by Campbell in 1832 ( 4 ). Previously, the disease was classified as acute/subacute/chronic based on the time of clinical presentation and outcomes. The HP guideline was updated in 2020 based on a combination of international clinical experience and available evidence by experts of the American Thoracic Society (ATS), the Japanese Respiratory Society (JRS), and the Latin American Thoracic Society (ALAT).

A recent classification of HP into non-fibrotic and fibrotic subtypes has helped to fill important gaps in the optimal diagnostic approach and disease classification ( 3 ).

Despite the recent data and guidelines suggesting new approaches for HP assessment, there are still significant gaps in the epidemiology, pathogenesis, diagnosis, and management of occupational HP. In this review, current information about occupational HP is discussed. A detailed literature review was conducted with a general search method using the keywords “occupational hypersensitivity pneumonia” and “hypersensitivity pneumonia” in PubMed, Google Academic, Medline, and Elsevier Science Direct databases. As a result, information about occupational HP will be discussed in line with the current literature.

### Epidemiology

The incidence and prevalence of HP vary with climate, environmental, or occupational exposures. However, the lack of accepted diagnostic criteria has made it difficult to determine the incidence and prevalence of HP ( 4 ). While the incidence of HP is 0.3-0.9 per 100.000 in the general population, the incidence of occupational HP was reported as 1.4 per 100.000 in the surveillance of work-related and occupational respiratory diseases between 1996 and 2015 ( 5 ). Although HP can occur at any age, patients are most commonly diagnosed in their 50s or 60s. The gender distribution is almost equal; however, the chronic form can be seen more commonly in males ( 6 ).

HP typically develops as a result of repetitive exposure to household, occupational, or environmental factors, although the specific factor contributing to disease development is often unidentified. Previous research has suggested a relationship between exposurespecific factors, such as concentration, duration, frequency of exposure, particle size, particle resolution, and the clinical course of the disease. However, this relationship was not clearly defined in the ATS/JRS/ALAT guideline ( 3 ). Occupational factors causing HP are classified as bacteria, fungi, enzymes, animal and insect proteins, low molecular weight chemicals, and plant proteins (Table 1) ( 1 , 7 ).

Farmer’s lung is known as the first described type of HP. The disease develops with the inhalation of microorganism antigens found in straw and grain warehouses with high humidity. The most commonly described agents are Thermophilic actinomycetes, Saccharopolyspora rectivirgula, Thermoactinomyces viridis, and Thermoactinomyces sacchari ( 4 ). Bird fancier’s lung is another common type of HP. Chemical-induced HP is rare. It is thought that methyl acrylate and acrylate compounds, which dentists substitute for amalgam fillings, lead to an increased incidence of HP in dentists ( 4 ). Anhydrides used in plastics, paints, resins, and adhesives, as well as other compounds such as triglycidyl isocyanurate, often used in paints, are also considered to cause HP ( 4 , 8 ).

New triggers for occupational hypersensitivity have recently been identified, including styrene in plastic and rubber production, Aspergillus oryzae, which causes hypersensitivity reactions, and fluorocarbon resin spray used for waterproofing leather and suede ( 5 ). In addition, metalworking fluids and Mycobacterium immunogenum, an atypical Mycobacterium commonly found in metalworking fluids, are also linked to HP ( 4 ). As more triggers are discovered, clinicians can more easily diagnose the condition.

**Table 1:** Principal agents causing occupational hypersensitivity pneumonitis ( 1 , 7 )

Category	Agents	Occupational areas and/or occupational exposure
Bacteria	Thermophilic actinomyces	Farmers, sugarcane workers, cap mushroom workers, organic fertilizer workers, ventilation system
Bacteria	Lichtheimia corymbifera	Farmers
Bacteria	Acinetobacter, Ochrobactrum	Metal working fluids
Bacteria	Streptomyces albus	Compost workers
Bacteria	Klebsiella oxytoca	Humidifiers
Bacteria	Bacillus subtilis enzymes	Detergent industry
Bacteria	Mycobacterium avium complex and other nontuberculous mycobacteria	Spa workers (Hot-tub lung)
Bacteria	Mycobacterium immunogenum	Metal working fluids, machine operators
Fungi	Apergillus spp	Tobacco growers, malt workers, fertilizer’s lung
Fungi	Alternia alternata	Humidifiers, wood workers
Fungi	Botrydis cinerea	Wine makers
Fungi	Cladosporium spp	Spa workers
Fungi	Penicillium frequentens	Suberosis
Fungi	P. caseii, P. Roqeforti	Cheese workers
Fungi	P. brevicompactum, Fusarium	Farmers
Fungi	Pleurotus osteatus	Mushroom workers
Fungi	Trichosporum cutaneum, T. ovoides	FSummer-type HP
Fungi	Candida spp	Saxophone lung
Animal & insect protein	Avian serum and feather proteins	Bird breeders
Animal & insect protein	Cat feather and animal fur	Furrier’s lung
Animal & insect protein	Mollusk Shell	Nacre industry
Animal & insect protein	Silk	Textile workers
Animal & insect protein	Rat serum proteins	Laboratory workers
Animal & insect protein	Sitophilus granarius	Farmers
Plant proteins	Coffee/tea dust	Food processors
Plant proteins	Legumes, malt	Food processors
Low molecular weighted chemicals	Diisocyanate	Chemical and polyurethane industry, painters, plastic workers
Low molecular weighted chemicals	Acid anhydrides	Plastic workers
Low molecular weighted chemicals	Acrylate compounds	Dental technicians
Low molecular weighted chemicals	Pharmaceutical agents: penicillins, cephalosporins	Pharmaceutical industry
Enzymes	Phytase, subtilisin, amylase	Animal feeding, cleaners
Metals	Cobalt	Hard-metal workers
Metals	Zinc	Smelters
Metals	Zirconium	Ceramic workers

#### Pathophysiology

HP is an inflammatory disease that develops due to type 4 (T lymphocyte-mediated) and type 3 (immunocomplex-mediated) hypersensitivity reactions, that cause sensitization as a result of repetitive antigen exposure. As a result of the type 3 hypersensitivity reaction being triggered, high levels of specific IgG precipitating antibodies are produced in the serum, while continuous antigen exposure triggers the type 4 hypersensitivity reaction that stimulates CD8 T lymphocytes, leading to macrophage activation and granuloma formation ( 9 ). While nonspecific inflammation develops in the early stages of exposure, lymphocytic inflammation and non-caseating granulomas in the center of the bronchioles develop with the progression of the disease, and bronchiolitis obliterans and fibrosis develop in the later stages of the disease ( 7 , 10 ).

Genetic factors are thought to play a role in the development and progression of HP. However, specific genetic factors that increase individual susceptibility have not yet been identified. The manifestation of different clinical presentations among individuals exposed to the same agent suggests a possible genetic link with MHC class II, particularly HLA-DR, and DQ ( 9 ). Studies have also found that HP patients are more likely to have short telomere lengths and minor alleles of the Mucin 5B (MUC5B) gene rs35705950, which are associated with fibrosis extent ( 11 ). Short telomere length has also been linked to the usual interstitial pneumonia (UIP) pattern, which presents with diffuse fibrosis and reduced survival ( 11 , 12 , 13 ).

##### Clinical Course and Current Classification

Previously, HP was classified as acute, subacute, or chronic, depending on the duration of the disease. In practice, classifying the disease in this way has proven difficult, and it has been found to have limited prognostic value. Therefore, based on subsequent studies and new information obtained from imaging, bronchoalveolar lavage (BAL), and clinical findings, a new classification has been proposed that correlates with radiology, clinical presentation, and histopathology. Accordingly, HP was classified as non-fibrotic HP and fibrotic HP ( 3 , 14 ).

HP can present as an acute disease, or it may be detected during the fibrosis stage or an acute exacerbation ( 14 ). Symptoms such as dyspnea, cough, chest pain, weight loss, and low-grade fever may be observed in both subtypes of HP. However, fever, malaise, cough, and sputum may also be seen after intense exposure, and this condition complicates the differential diagnosis of HP ( 6 ). Symptoms may begin acutely within days or weeks or may develop over months or years. Although it is not specific to HP, a physical examination may reveal rales, club fingers, and cyanosis. However, in the presence of fibrosis, hearing an inspiratory rhonchus (squeak) on auscultation is characteristic ( 6 ).

In patients diagnosed with non-fibrotic HP, improvement may be observed in clinical and radiological findings when exposure is eliminated. However, in fibrotic HP, especially if the UIP pattern has developed, the prognosis of the disease is poor ( 3 ). The poor prognostic factors of the disease are smoking, low initial vital capacity, absence of lymphocytosis in the bronchoalveolar fluid, presence of interstitial pneumonia, continuous exposure to the triggering factor, and failure to identify the cause ( 15 , 16 , 17 ).

###### Diagnosis

HP has variable clinical features that may differ from patient to patient. As a result, patients may be misdiagnosed with either a respiratory tract infection or idiopathic interstitial pneumonia (IPF) ( 18 ). There is no gold standard diagnostic method to help diagnose HP. The most critical aspect of diagnosing HP is for the clinician to maintain a healthy level of skepticism and take a thorough history of exposure ( 14 , 18 14,18 ). In addition, specific IgG antibodies, lymphocyte level in BAL, high-resolution computed tomography (HRCT) evaluation, and if necessary, histopathological sampling should be performed. Specific inhalation testing may help diagnose HP, confirm its etiology, and determine whether a biopsy is necessary ( 14 ).

Clinical findings have an acute onset, and agent detection is possible in non-fibrotic HP.

In non-fibrotic HP, the presence of centrilobular nodules in HRCT and lymphocytosis in BAL support the diagnosis. Conversely, in fibrotic HP, clinical findings may have occurred later, and the causative factor may be difficult to determine. However, fibrotic HP is confused with fibrotic lung diseases of unknown cause, such as IPF ( 3 ). Therefore, cases of suspected HP should be evaluated collaboratively by clinicians, radiologists, and histopathologists ( 3 , 19 ). In cases where occupational HP is suspected, the multidisciplinary council may seek the advice of an occupational diseases specialist. Occupational HP should be suspected when a patient describes symptoms that worsen during work and improve when away from work. Therefore, in order to arrive at the correct diagnosis, it is essential to inquire about exposure and symptoms. When taking an occupational history, healthcare providers should ask about the patient’s previous and current jobs, the types of dust, aerosols, and chemicals they were exposed to, the use of protective equipment, and the workplace ventilation. Additionally, obtaining material safety forms and evaluating the work area can also help with the diagnosis ( 20 ).

####### Determination of the Triggering Antigen

HP is an immune-mediated lung disease that develops after exposure to antigens in susceptible individuals. Identifying the specific antigen is crucial for diagnosing the disease and has been shown to impact disease prognosis, recurrence, and quality of life ( 21 , 22 ).

A study found that the average survival rate for patients with chronic HP was 8.75 years when the triggering antigen could be identified, compared to 4.88 years when it could not. Identifying the antigen allows for earlier diagnosis and treatment by avoiding exposure, leading to a better prognosis ( 22 ).

Thoroughly questioning the history of exposure is crucial in identifying the potential causative agent during the assessment and diagnosis of HP.

Occupational hygienists can conduct occupational and environmental sampling of industrial and agricultural areas where animal and plant products, microbial bioaerosols, and metal or chemical compounds are involved in the etiology of HP. This sampling can be useful in detecting antigens ( 23 ). Although measuring the antigen exposure level in the workplace can provide insight into how to detect the suspected agent, it is not possible to estimate the threshold value of the agent for HP. This is because the disease develops through a susceptibility mechanism that exhibits individual differences ( 19 ).

Antigen detection is difficult due to the lack of a standardized laboratory test. Specific IgG in serum, specific inhalation testing, antigen avoidance testing, and lymphocyte proliferation testing help the diagnosis, yet have limited clinical use.

One of the methods to help identify the triggering agent is the measurement of specific IgG in serum, which indicates immunological sensitization due to antigen exposure. In cases with suspected occupational HP, the detection of a high level of specific IgG against an identifiable allergen in the workplace is strong evidence of occupational etiology ( 11 ). In addition, a decrease in the level of specific IgG with avoidance of the antigen is also a finding that supports the diagnosis ( 1 ). However, the efficacy of this test is limited because most of the occupational HP agents cannot be identified, and antigenic preparations cannot be prepared ( 19 ). Specific IgG tests should be evaluated in conjunction with other diagnostic tests included in the HP diagnostic algorithm ( 23 ).

The specific inhalation test is another helpful test in the diagnosis of HP, which is based on the controlled exposure of the patient to the suspected antigen and then monitoring the physiological parameters. While there are standardized procedures for specific inhalation tests for occupational asthma, there is not yet a standard procedure for HP. The test is performed either by applying the antigen solution as a nebulizer aerosol or by exposing the patient to the agent in their natural or laboratory environment. The test should be performed by specialized centers, and the patient should be closely monitored during the test. This test is not recommended for patients who have severe functional limitations. There is no consensus on the role of the specific inhalation test in the HP diagnostic algorithm, how to prepare antigen solutions, or the factors that define a positive test result ( 23 , 24 ). Some specialized centers consider the test positive if there is more than a 15% reduction in forced vital capacity (FVC) and more than a 20% reduction in lung diffusing capacity of carbon monoxide (DLCO), or if clinical symptoms (dyspnea, cough, desaturation, hyperthermia) and radiological changes are present (25). The benefit of the specific inhalation test is not clear as there may also be falsenegative or false-positive results. However, in a patient with ILD, specific inhalation test positivity increases the probability of the diagnosis of HP and may reduce the need for invasive procedures such as surgical biopsy ( 23 , 24 ).

The lymphocyte proliferation test (LPT) is another laboratory test that aims to evaluate antigen sensitivity and is used in the diagnosis of HP resulting from inhalation of bird proteins. However, the LPT has not been standardized and is not widely used ( 26 ).

An antigen avoidance test is performed to evaluate the effect of antigen avoidance on clinical response.

The results of this test are indicative of HP if there is an improvement in vital capacity, Krebs von den Lungen 6 (KL-6), white blood cell count, and body temperature measurements at the end of two weeks without exposure compared to baseline ( 27 ).

Although clinical improvement is observed with antigen avoidance in patients with non-fibrotic HP, the benefit of antigen avoidance in patients with fibrotic HP is limited (25). Also, it is a helpful test in the differential diagnosis of IPF and fibrotic HP, which are often confused in the differential diagnosis. However, this test is still under study and more research is needed ( 27 ).

######## Bronchoalveolar Lavage

BAL findings of HP are consistent with lymphocytic alveolitis ( 7 ). The American Thoracic Society published guidelines in 2012 on the clinical efficacy of BAL fluid in interstitial lung disease. Here, the role of BAL fluid analysis in the diagnosis and treatment of ILD was investigated and recommendations were made. Accordingly, in BAL, lymphocyte levels were found to be 25% and higher in granulomatous diseases (sarcoidosis, HP, chronic beryllium disease), and over 50% in HP and nonspecific interstitial pneumonia (NSIP) ( 28 ).

In a recent meta-analysis, the role of the lymphocyte ratio in BAL in the diagnosis of chronic HP was investigated.

This study suggests that the degree of alveolar inflammation in HP is influenced by the extent of fibrosis, the type of exposed antigen, and the duration since the last exposure. The percentage of lymphocytes in the inflammation decreases as the disease progresses ( 29 ).

In a multicenter study investigating the cell distribution in BAL in HP and its contribution to the diagnosis, the total cell count and lymphocyte ratio were found to be high in BAL. Furthermore, the lymphocyte level was found to be significantly lower in the chronic stage compared to the acute and subacute stages, when considering cell distribution according to the disease stage ( 30 ).

In HP, CD4/CD8 ratio decreases in BAL due to CD8 + T cell dominance. While CD4/CD8 can be at different levels in different forms of HP, it also varies according to smoking status, type of inhaled antigen, and duration of exposure ( 31 , 32 , 33 ).

######### Radiology

Radiological evaluation is important in the diagnosis of HP ( 27 ). Chest X-ray is usually normal or there are nonspecific findings. Therefore, HRCT is the most useful radiological method in the diagnosis of HP.

Some radiological features have been found to have high specificity for HP. The characteristic pattern and distribution of findings in HRCT are useful in distinguishing chronic HP, NSIP, and IPF. The radiological image of HP may be correlated with the histopathological stage at the time of diagnosis. While HRCT is very useful for the diagnosis of HP, it should not be used alone in the differential diagnosis or definitive diagnosis ( 11 ).

While HP was previously classified as acute, subacute, and chronic forms, in the 2020 ATS/JRS/ ALAT guideline it was recommended to be done as fibrotic HP and non-fibrotic HP by the committee (3). As stated in the guideline, heterogeneous attenuation in which mosaic attenuation, mosaic perfusion, air trapping, and three-density pattern areas can be seen together is dominant in HP radiology. A new term, “three-density pattern” (headcheese sign), refers to sharply separated normal areas, high-density groundglass areas, and low-density areas that can be observed in inspiratory HRCT, and this finding is specific for fibrotic HP ( 3 , 15 ).

While the radiological features of fibrotic and nonfibrotic HP may differ, both subgroups were classified as “typical for HP”, “compatible with HP” and “indeterminate HP” with the recommendation of the committee ( 3 ).

########## Radiological Features of Non-Fibrotic HP

Typical HRCT findings in HP include mosaic attenuation, ground-glass opacity, and at least one finding suggestive of small airway disease (<5 mm centrilobular nodule with indistinct borders on inspiratory HRCT). This pattern is usually bilateral and symmetrically distributed, located both axially and craniocaudally. The presence of uniform and thin ground-glass nodules (GGO), consolidation of air trapping, and presence of lung cysts are other features. Although these features are not specific to non-fibrotic HP, they may be indicative of nonfibrotic HP when evaluated in conjunction with the appropriate clinical features ( 3 ).

The coexistence of pulmonary fibrosis and bronchial obstruction are findings suggestive of fibrotic HP. Pulmonary fibrosis in HP most commonly presents either alone or as traction bronchiectasis in areas of ground-glass opacity, septal thickening, and fine and coarse reticulation. In severe fibrotic forms of HP, honeycomb formation may accompany. The most common locations for HRCT findings in HP are the middle or mid-lower zones, although they may be equally distributed across all zones where the basal regions are relatively preserved. As in non-fibrotic HP, centrilobular nodules and mosaic attenuation may also be seen. Three density patterns (groundglass opacity, decreased attenuation, and vascularized lobules) are specific HRCT findings for fibrotic HP and may be accompanied by normal areas ( 3 , 34 ).

Since chronic or fibrotic HP imaging includes a combination of different radiological partners, it is difficult to diagnose and differentiate it from interstitial lung diseases that progress with fibrosis, especially UIP and NSIP ( 34 ).

########### Histopathological Features

The biopsy requirement is still valid for the definitive diagnosis of HP, which is diagnosed clinically, radiologically, and pathologically with a multidisciplinary approach. However, recently, less invasive approaches are preferred instead of invasive ones. The biopsy is the gold standard in patients who could not be diagnosed with radiology and clinical findings.

Given the heterogeneous histological features of HP, a surgical method that provides a larger tissue sample is preferred in order to detect the most characteristic features. Transbronchial biopsies and transbronchial cryobiopsies have been suggested as alternatives ( 35 ). While the biopsy is usually not necessary in the acute period, multiple biopsies may be necessary for the diagnosis of fibrotic HP, especially in the differential diagnosis of IPF ( 36 ).

The three typical histopathological findings of the disease are peribronchial diffuse interstitial inflammatory infiltrates, chronic bronchiolitis, peribronchiolar nonnecrotizing granulomas, and UIP may develop in the later stages of the disease. The UIP pattern, fibrotic NSIP, and the presence of airway-centered fibrosis have a worse prognosis than cellular NSIP or peribronchial inflammation accompanied by poorly constructed granuloma ( 37 ). According to the new consensus, non-fibrotic HP and fibrotic HP has been classified as “typical for HP”, “probable HP” and “indeterminate HP” ( 3 ).

############ Histopathological Features of Non-Fibrotic HP

In the histopathological diagnosis of non-fibrotic HP, cellular interstitial pneumonia, cellular bronchiolitis, weak or loosely formed granulomas, and/or organized pneumonia are typical findings. Interstitial inflammation and/or randomly distributed multinucleated giant cells within the bronchiolar walls may accompany these findings ( 3 , 6 ).

############# Histopathological Features of Fibrotic HP

Histopathological features may be similar to other interstitial lung diseases. In order to make a differential diagnosis and especially to differentiate it from IPF, biopsy samples should be taken from more than one region. Findings that support fibrotic HP are chronic fibrosis or bronchiolocentric fibrosis, bronchiolar epithelial hyperplasia, and the presence of poorly structured non-necrotizing granuloma ( 3 , 6 ).

############## Pulmonary Function Tests

Although pulmonary function tests (PFTs) may be normal in the acute phase of HP, as fibrosis develops, restrictive findings are most commonly seen in PFTs. The decrease in carbon monoxide diffusion may be accompanied by an increased arterial-alveolar oxygen gradient or exercise dyspnea ( 31 ).

Improvement in these findings may be observed during the acute phase of HP following treatment or avoidance of exposure, whereas the disease may become irreversible as it progresses to the fibrotic form, which is a significant determinant of mortality ( 15 , 19 ).

In the presence of concomitant emphysema and/or bronchiolitis, an obstructive pattern may be seen in PFTs due to increased residual volume. In those working in risky occupations (such as metalworking fluid exposure), the differential diagnosis of occupational asthma and HP should be made in terms of increased airway sensitivity. Observing an increase in PFTs performed after exposure was avoided compared to PFTs performed in the working environment is an objective finding for the diagnosis of occupational HP ( 11 ). Functional impairment may not always be correlated with the severity of radiological abnormalities. PFTs are important in determining the severity of the disease at the time of diagnosis and in the follow-up of the disease ( 31 ).

############### Diagnostic Criteria

Important criteria in the diagnosis of HP are the description of exposure (detailed history and exposures in the workplace, antigen-specific IgG, specific inhalation test), imaging method, ymphocytosis in BAL, and histopathological findings ( 3 ).

According to the recommendations of the ATS/JRS/ ALAT guideline, in a suspected case of HP with interstitial abnormalities on thorax imaging, exposure history or presence of specific antigen in serum, imaging findings in HRCT, presence of lymphocytosis in BAL should be investigated and histopathological sampling should be performed when necessary. In cases with uncertainty in diagnosis, multidisciplinary evaluation is recommended. The aim is to reach a diagnosis through less invasive interventions ( 3 )

################ Treatment

There is currently no universally approved approach for the treatment of HP ( 38 ). The primary step in treating HP is to end the patient’s exposure to the triggering antigen. Early detection and avoidance of the triggering antigen that the patient is exposed to have prognostic importance. Complete avoidance may not always be possible due to being unable to detect suspected antigens and economic, social, or occupational reasons ( 9 , 34 , 39 ). However, the disease may progress even with complete avoidance ( 34 , 40 , 41 ).

If the factor causing HP is not detected and exposure continues, the disease becomes progressive. Pharmacological treatments are needed if the disease persists despite avoidance of exposure or in the presence of progressive disease. In the acute phase of the disease, nonspecific symptoms such as cough, shortness of breath, and flu-like symptoms may resolve spontaneously within days or weeks after exposure is discontinued. However, in patients who do not show clinical improvement, corticosteroid therapy is given ( 38 ). Corticosteroids remain the mainstay of pharmacological treatment, but there are no adequate studies on the optimal dose or duration of use, yet ( 42 ). Cases with ground-glass opacity indicating active inflammation on HRCT, lymphocytosis (>20%) in BAL, and/or histopathologically cellular interstitial inflammation or granulomatous inflammation are more likely to respond to corticosteroid therapy, compared to those without these findings ( 26 , 40 ).

In a cohort study investigating the effects of avoidance of exposure and corticosteroids on both two subgroups of the disease, there were positive effects of corticosteroid treatment on the decrease of FVC and DLCO in non-fibrotic HP patients, while it was not found to be effective in fibrotic HP patients.

Moreover, no effects on survival were detected in either of the subgroups. Avoidance of exposure has positive effects on FVC and DLCO in non-fibrotic HP patients and positive effects on FVC in fibrotic HP patients, but no effect was detected on survival in either group ( 43 ).

Immunosuppressive treatments such as methotrexate, mycophenolate mofetil (MMF), and azathioprine (AZA) may be preferred in patients with chronic and progressive disease, who cannot use corticosteroids due to side effects, or while reducing the dose of corticosteroids ( 9 ). In a retrospective study by Morisset et al., a positive effect on DLCO was observed in each regimen in patients treated with MMF and AZA, but no positive effect was observed on prognosis ( 44 ). In the study of Adegunsoye et al., no effect of AZA or MMF was observed on pulmonary functions ( 45 ).

The survival rate of chronic HP patients requiring immunosuppressant therapy is generally low. Noncorticosteroid treatments have been introduced to avoid the side effects of corticosteroids and reduce the frequency of adverse effects. Rituximab can be used as a salvage therapy in cases of refractory chronic HP ( 35 ). In a retrospective observational study of 20 patients with chronic HP treated with rituximab, it was concluded that rituximab treatment resulted in a decrease in the rate of FVC reduction, with corticosteroid use being significantly decreased after initiation of rituximab ( 42 , 46 ).

The effects of immunosuppressive treatment in fibrotic HP are limited.

Given the similarity of the pathological mechanisms of fibrotic diseases, it has been suggested that antifibrotic treatments may also be effective in managing HP with a fibrotic phenotype. IPF, the leading type of progressive fibrosis, and fibrotic HP are similar in terms of radiological, histopathological, and clinical features.

The use of anti-fibrotic agents, such as pirfenidone and nintedanib, which have demonstrated efficacy in IPF, is limited in the treatment of fibrotic HP and is primarily reserved for research studies. The INBUILD study was conducted to investigate the efficacy of nintedanib in cases of non-IPF pulmonary fibrosis. In the study, 663 patients with fibrosis affecting more than 10% of the lung volume on HRCT were included and 26% of them were chronic HP patients. A study has shown that nintedanib significantly reduced the annual decline in FVC compared to placebo, although diarrhea and liver function test abnormalities were observed more frequently in patients treated with nintedanib compared to placebo ( 47 ). Anti-fibrotic treatment is promising for chronic HP, but more randomized controlled trials are needed to determine the time to start treatment, the benefits of combined immunosuppressive and anti-fibrotic therapy, and their potential effects in first-line therapy for chronic HP ( 19 , 47 ).

In HP cases with progressive disease despite treatment, early evaluation for transplantation and palliative care when necessary should be performed ( 48 ).

################# Prognosis

Occupational HP can result in a full recovery after an acute attack, or it can lead to different clinical outcomes such as the progression to progressive pulmonary fibrosis. The prognosis may vary depending on the disease subtype, duration and intensity of exposure, antigen type, histopathological type, and genetic predisposition. The presence of radiological or histopathological fibrosis is a poor prognostic factor ( 31 , 49 ). If the disease is diagnosed and treated in the acute period, the prognosis is generally good. In cases where HP is diagnosed at an advanced stage, particularly after fibrosis has developed, the disease tends to be progressive and can be fatal ( 32 ). In some cases, occupational HP may progress badly if the allergen factor in the workplace cannot be determined or if the person continues to work in the same environment due to socio-economic conditions ( 11 ).

Despite antigen avoidance and treatment, some patients may experience disease progression. The reason for this has not yet been explained ( 50 ). The frequency of acute attacks in chronic HP is another factor associated with poor prognosis

In one study, it was observed that 14 out of 100 patients with chronic avian and farmer’s lung experienced an acute exacerbation, and among them, 12 patients died ( 31 , 51 ). In a study by De Sadeleer et al., low BAL lymphocytosis (<20%) and the presence of honeycomb on HRCT were found to be associated with poor ten-year survival and poor response to corticosteroids. In contrast, in patients without honeycomb and high BAL lymphocytosis, the FVC response was evident with initiated corticosteroids ( 52 ).

In addition to these, advanced age, male gender, low functional status at baseline, undetected antigen, and presence of comorbidities are other prognostic factors ( 53 ).

################## Prevention

As prevention of exposure is the major determinant of the prognosis of HP, detection of the agent has great importance.

Occupational hygienists can determine risky allergen sources by conducting workplace visits, analyzing air and surface samples, and implementing cleaning and safety measures. In addition, removing employees diagnosed with HP from the workplace, screening other employees, and providing health and safety training can help prevent the development and progression of the disease ( 11 ).

## CONCLUSION

In conclusion, occupational diseases can be prevented. Identifying the factors that cause HP in the workplace is crucial for early diagnosis and prevention of disease progression. Due to the different phenotypes of HP, multidisciplinary evaluation is recommended for diagnosis. Although many studies have shown promise in treatment, further studies are necessary to clarify the safety profiles and efficacy of anti-fibrotic and immunosuppressive agents.
